# Breaking of Icosahedral Symmetry: *C*_60_ to *C*_70_

**DOI:** 10.1371/journal.pone.0084079

**Published:** 2014-03-05

**Authors:** Mark Bodner, Jiri Patera, Marzena Szajewska

**Affiliations:** 1 MIND Research Institute, Irvine, California, United States of America; 2 Centre de Recherches Mathématiques, Université de Montréal, Montréal, Québec, Canada; University College London, United Kingdom

## Abstract

We describe the existence and structure of large fullerenes in terms of symmetry breaking of the 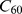 molecule. Specifically, we describe the existence of 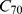 in terms of breaking of the icosahedral symmetry of 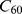 by the insertion into its middle of an additional 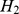 decagon. The surface of 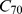 is formed by 12 regular pentagons and 25 regular hexagons. All 105 edges of 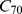 are of the same length. It should be noted that the structure of the molecules is described in exact coordinates relative to the non-orthogonal icosahedral bases. This symmetry breaking process can be readily applied, and could account for and describe other larger cage cluster fullerene molecules, as well as more complex higher structures such as nanotubes.

## Introduction

**Figure 1:**
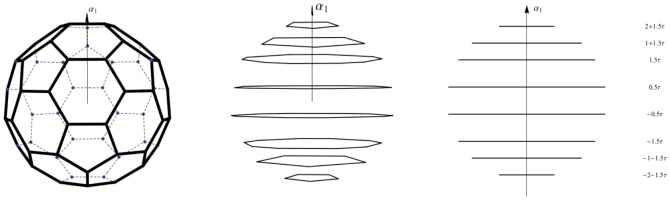
The structure of the *C*_60_ molecule. (a) The polytope 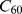 is formed by 60 vertices equidistant from its center. Its surface consists of 12 regular pentagons and 20 regular hexagons. All 90 edges are of the same length. (b) The 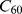 polytope viewed in the direction almost parallel to the plane spanned by 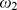 and 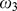, which makes the 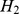 orbits (pentagons and decagons) easy to identify. (c) The 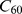 polytope viewed in the direction parallel to the 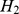 plane spanned by 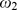 and 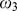. Surface edges of 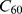 are omitted in (b) and (c). Horizontal segments are projections of the 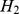 orbits. The number in a row shows the 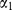 coordinate of the 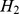 orbit. The vertical direction is that of 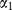.

**Figure 2:**
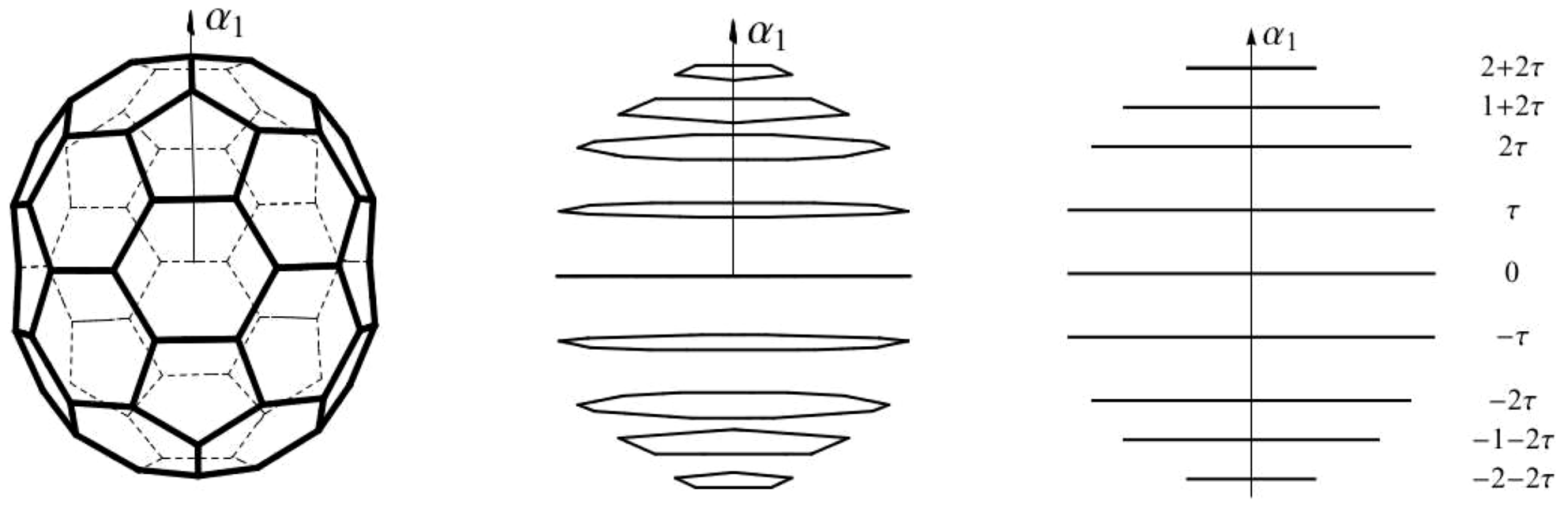
The structure of the *C*_70_ molecule. (a) The polytope 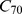 has 105 edges and 12 pentagonal and 25 hexagonal faces. (b) 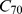 viewed from a direction almost parallel to the plane of 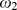 and 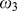. (c) The 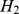 structure of 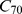 viewed from a direction parallel to the plane of 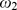 and 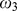. The column of numbers shows the 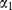-coordinate of the 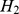 orbits of vertices of 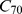. The inserted decagon has the 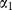-coordinate equal to 0. Surface edges are omitted in (b) and (c).

Fullerenes are molecules composed entirely of carbon, taking the form of a cage or tube. The family of cage cluster fullerenes is also commonly referred to as buckyballs. The most stable and commonly occurring member of this family is the molecule 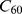, which consists of 60 carbon atoms arranged in a structure of truncated icosahedrons, made of hexagons and pentagons, with carbon atoms at the corners of each hexagon and a bond along each edge (creating the well-known soccer ball structure - Fig. 1). This structure has been investigated and determined experimentally in both the solid state [1] and in the gas phase [2]. The second most commonly occurring cage structure fullerene is the molecule 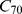, composed of 70 carbon atoms. Electron diffraction and theoretical studies have verified that this molecule possesses a “rugby ball” structure with a pinching of the waist as the bond lengths follow a simple pattern determined by their relationship to the 5- and 6-membered rings [2], [3] - Fig. 2.

Much effort has been directed to answer why the fullerenes 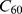 and 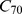 are so stable and which other higher fullerenes with different sizes and shapes can be formed as stable entities [2], [4]–[6]. With respect to the 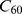 molecule, its stability and its position as the most commonly occurring buckyball can be attributed, at least in part, to its high degree of symmetry [7]. In addition to 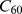 and 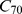, many other larger fullerenes have been observed, while theoretical calculations have indicated that all fullerenes with an even number of carbon atoms can exist [8]. Significant amounts of work have gone towards elucidating the structure of these higher fullerenes. For example, the structures of 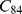, as well as 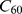 and 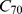 have been identified through spectroscopy studies [9]–[12], and by chemical derivatization [13], [14], while others have been proposed theoretically [15].

In the present work we consider the existence and structure of higher fullerenes as a symmetry breaking problem, starting from the 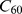 molecule which possesses the highest degree of symmetry. Guided by the common practices in particle physics, we consider the description specifically of the 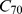 molecule as a symmetry breaking problem, with the additional twist that the usual branching rule for the icosahedral symmetry group 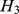 to the dihedral symmetry group 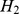 is enhanced by adding to it one more decagonal term. The group 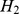 is the lowest noncrystallographic finite reflection group. We consider the icosahedral symmetry group 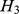 of order 120 of certain carbon molecules as the exact symmetry that is broken to its subgroup 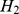 or order 10 dihedral symmetries. We also suggest within this framework how higher order structures such as nanotubes may naturally arise. This provides a framework for understanding of the observed even carbon number rule and for predicting higher order structures which may be assembled.

The paper is unique in providing exact coordinates of the vertices of the fullerenes thus eliminating any additional numbering conventions used elsewhere [16]. This opens the possibility of defining special functions of 3 variables generated by the vertices (see Example 3), to study their possible orthogonality, and conceivably even the corresponding orthogonal polynomials defined by the fullerene structures.

## Icosahedral bases in 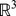


In order to get exact coordinates of polytopes related to icosahedral symmetry, one has to use bases in the real 3-dimensional space 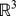 that reflects the symmetry, namely the simple roots 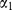, 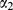, 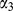 of the icosahedral group and their duals [17]. The geometric relations of the vectors in the 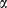-basis are described by the matrix of scalar products
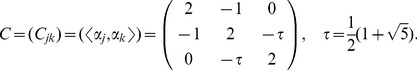
(1)The dual or reciprical 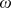-basis is defined by
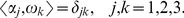
(2)The inverse matrix to 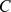 is calculated as follows,
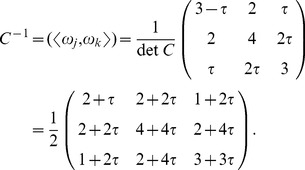
(3)


It follows from Eq.(2) that the 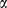
- and 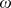-bases are related by the matrix equality 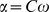, and 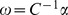. Explicitly we have
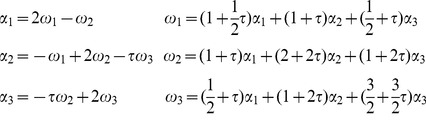
(4)


For the 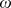-basis of 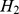 we choose 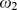 and 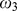 of 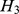. By Eq.(2), the direction orthogonal to the plane spanned by 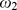 and 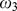, is that of 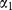.

The reflections 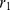, 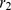, and 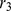 in 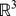 generate the icosahedral group of order 120. Their action on any vector 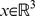 is given by
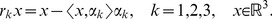
(5)In particular, 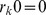 and 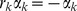, and also 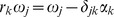.

## *C*_60_

Repeated application of the three reflections to the seed point 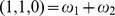 of 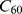, according to Eq.(5), yields the 60 vertices of 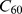 in the 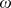-basis:



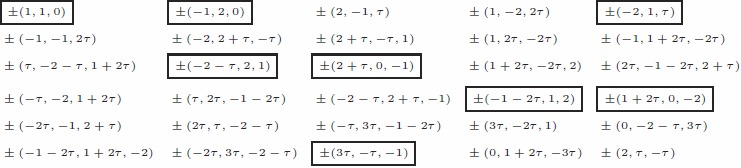
(6)


The points in which both the second and third coordinates are non-negative are the dominant points of the 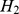 orbits (pentagons and decagons). Boxed points in which the second and third coordinates are positive dominate the decagons. The boxed points, which have 0 as their second or third coordinate, are the dominant points of 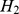 pentagons.

### Example 1

*The hexagon faces of* 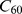 *come up naturally from the classification of its 2-faces* [*18*] *as one orbit of the seed hexagon. The symmetry group of the seed hexagon is generated by the reflections* 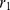 *and* 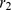 *. Similarly the pentagon faces of* 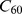 *come up naturally from the classification of its 2-faces as one orbit of the seed pentagon. The symmetry group of the seed pentagon is generated by the reflections* 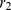 *and* 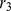 *.*

*Let us illustrate the construction of the seed hexagon and of the seed pentagon, starting from the dominant point of* 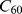 *:*

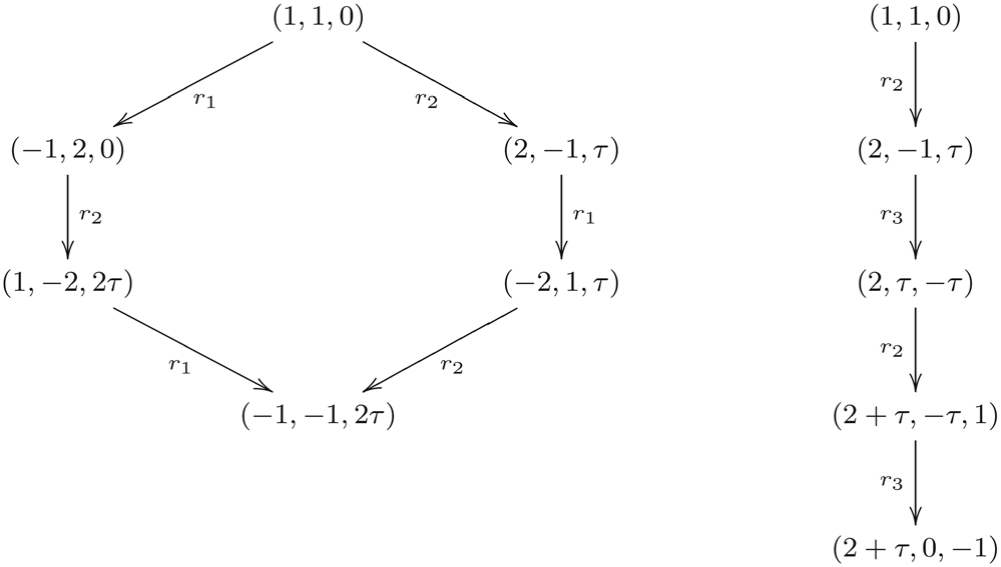



*The vertices of a hexagon and pentagon of the surface of* 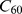 *adjacent to the dominant point of* 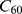 *are shown here in the* 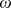 *-basis.*

### Example 2

*The three simple roots,* 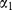 *,* 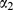 *,* 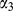 *, of the icosahedral group form a special basis in* 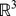 *. Their relative angles and lengths are read from the matrix* 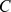 *(* *Eq.(1)* *). Those values define the icosahedral group* 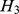 *. Therefore they take special positions also in* 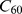 *.*

*Let us show that (i) the straight line containing* 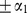 *passes through the center of opposite pentagons on the surface of* 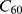 *. To show it, one needs to take the hexagon generated by* 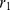 *and* 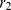 *, and add its vertices to verify that coordinates of the sum are zero in the plane spanned by* 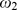 *and* 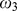 *.*

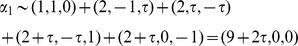

*(ii) The straight line containing* 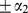 *passes through the center of opposite edges on the surface of* 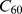 *that separate two hexagons.*

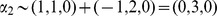

*(iii) The straight line containing* 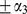 *passes through the center of opposite hexagons on the surface of* 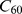 *.*

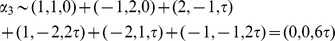



### Example 3

*In this example let us view each point* 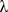 *of* *Eq.(6)* *as an exponential function,* 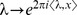 *, where* 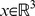 *, and then add up all 60 such exponentials. Call such a sum* 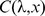 *. Since each* 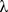 *comes with both signs in* *Eq.(6)* *, we have* 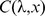 *equal to the sum of 30 cosines* 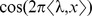 *. Properties of* 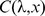 *deserves further study. The function ‘remembers’ the structure of* 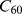 *in the entire 3-space and shows intricate interferences of the cosines with a clear maximum when* 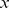 *is at the origin of* 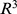 *. On the spherical surface of the* 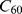 *shell, the function* 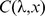 *depends periodically on the radius of* 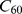 *.*

## *C*_70_

**Figure 3:**
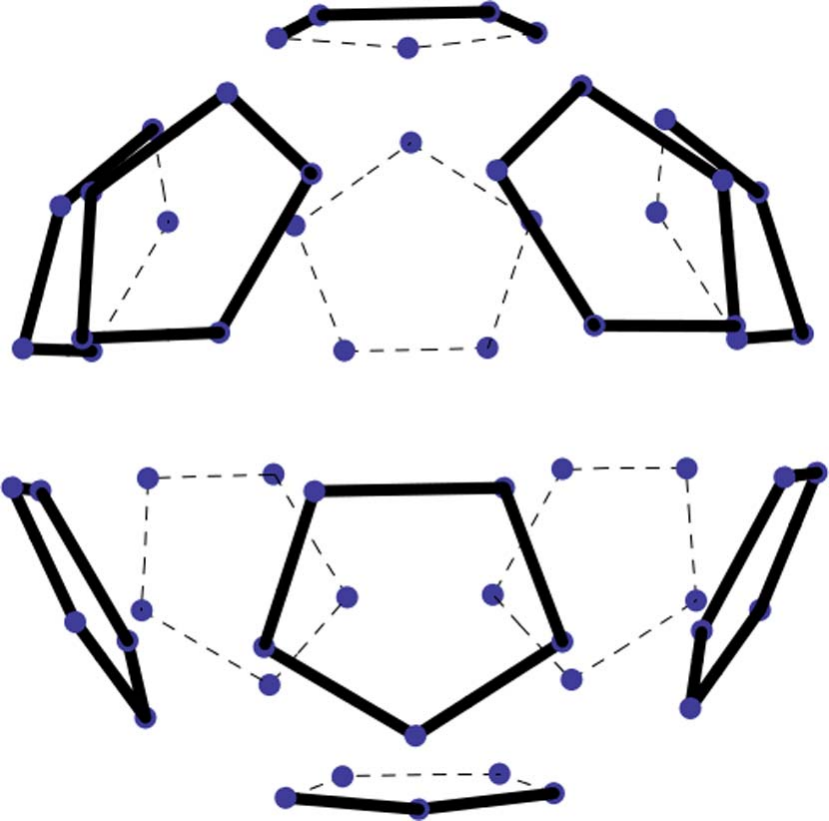
The 12 pentagons of the surface of 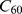
 **are shown without the hexagons.** The 60 dots are the vertices of 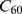. The polytope is oriented as in Fig. 1.

**Figure 4:**
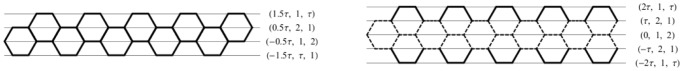
A ring of hexagons from the middle of the surface of 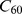
 **and** 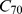 **unwrapped into the plane.** Horizontal lines indicate positions of the four 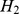 decagons in 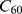 and five 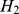 decagons in 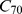. The dominant points identify the 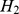 decagons relative to the basis 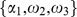. Dashed lines are the boundaries of the ring of five inserted hexagons.

The general idea, we pursue here for the modification 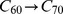, is first to decompose 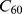 into the sum of 8 orbits of 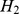, then to insert another 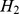 decagon into its middle. In Fig. 3 it can be seen that the upper and lower half of 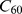 are connected by a ring of 5 hexagons. Replacing that ring by a larger one that is made out of 10 hexagons (see Fig. 4), one gets the polytope 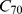. It is shown in Fig. 2 in three different views analogous to the presentation of 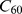 in Fig. 1.

### Symmetry breaking

The 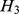 symmetry gets broken when the additional decagon is inserted into the middle of its decomposition into 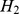-orbits. The 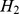 symmetry remains exact.
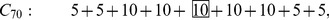
(7)The enlarged structure is 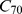 which has lost the spherical symmetry of 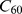. It has 70 vertices, and in the middle of it there are 5 consecutive parallel decagons centered at the 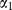-axis.

There are still two questions to be answered however before one can call it 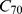. The answers to these questions must assure that the exterior surface of 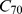 is composed of pentagons and hexagons of the same size as it is for 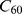. From Fig. 3 we see that the upper and lower halves of 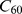 are bound by a ring of hexagons. (i) What are the distances between the five decagons, and (ii) what is the orientation of the inserted decagon in the 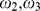 plane?

The answers to the questions are found by making two observations from Fig. 4, where the additional decagon is placed in the middle, so that its 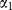 coordinate is zero.

- In order to keep the distances between the five decagons of 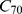 equal to what they are in 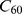, we have to shift correspondingly the upper and the lower halves of what used to be 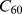. Their 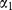 coordinates are increased and decreased by 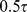 respectively.
- The first row of hexagons in Fig. 4 (right). belonged to the upper half of 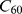. The second row in Fig. 4 (right) is situated as was the second row in Fig. 4 (left). There it was the top row for the lower part of 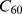. In Fig. 4 (right) it is the inserted middle row of 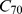. The third row of hexagons in Fig.4 (right) is the top row of the lower half of 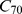. The third row of hexagons in Fig. 4 (right) is the top row of the lower half of 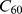. However, its position matches the hexagons of the first row. Hence the dominant points of the first and third rows differ by the sign of the first coordinate only.

Summarizing, below are the exact coordinate of the 70 vertices of 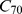 in the basis 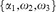:
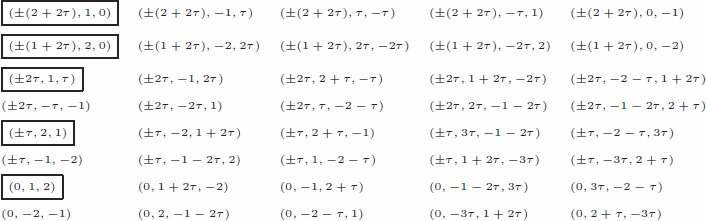
(8)


According to [19] the carbon polytope 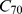 is slimmer in the middle. Such data can be matched by choosing a smaller radius for the decagon 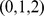 in Eq.(8) in the middle of 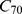. Also the edges leading to that decagon may have to be changed correspondingly. The boxed points in Eq.(8) are the dominant points.

In the present work we have described the existence of the molecule 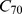 in terms of a symmetry breaking process of the insertion of an 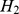 decagon (or equivalently inserting a ring of surface hexagons), thus breaking the icosahedral symmetry of 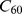. There is nothing to prevent however, the insertion of three or more rings of hexagons into the 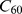 structure creating ones that are larger and more complex. Thus the mechanism enables the creation from 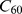, the fullerenes 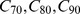 and so on. From the continuation of the process of the insertion of hexagon rings in this fashion, it can also readily be seen that it enables the creation of nanotubes of any length.
